# Monitoring daytime differences in moderate intensity exercise capacity using treadmill test and muscle dissection

**DOI:** 10.1016/j.xpro.2021.100331

**Published:** 2021-02-05

**Authors:** Yaarit Adamovich, Saar Ezagouri, Vaishnavi Dandavate, Gad Asher

**Affiliations:** 1 Department of Biomolecular Sciences, Weizmann Institute of Science, 7610001 Rehovot, Israel

**Keywords:** Metabolism, Model organisms

## Abstract

There is growing interest in medicine and sports in uncovering exercise modifiers that enhance or limit exercise capacity. Here, we detail a protocol for testing the daytime effect on running capacity in mice using a moderate intensity treadmill effort test. Instructions for dissecting soleus, gastrocnemius plantaris, and quadriceps muscles for further analysis are provided as well. This experimental setup is optimized for addressing questions regarding the involvement of daytime and circadian clocks in regulating exercise capacity.

For complete details on the use and execution of this protocol, please refer to Ezagouri et al. (2019).

## Highlights

• Exercise capacity is influenced by the time of day
• Protocol for determining moderate intensity exercise capacity using treadmill test
• Instructions for muscle dissection

## Graphical Abstract

**Figure:**
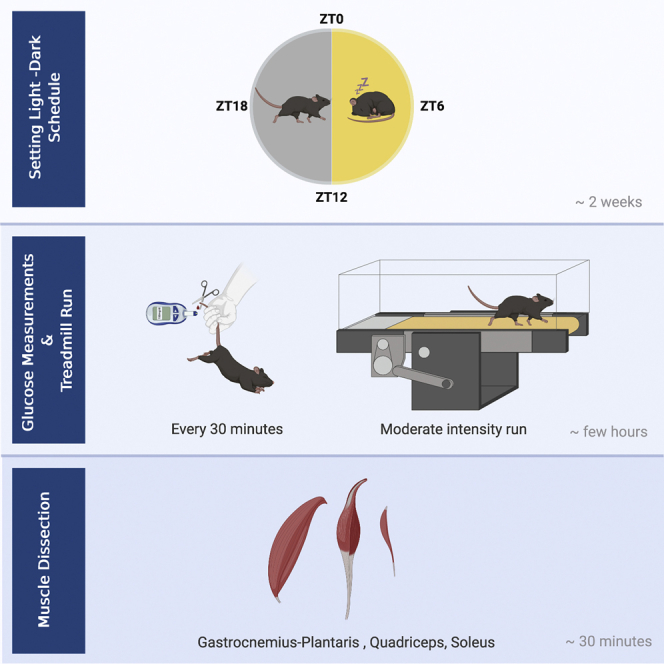

## Before you begin

### The importance of daytime to your experimental design

Testing endurance capacity in mice is often performed by allowing mice to run on a rodent treadmill and monitoring their running duration. Due to practical reasons, these studies are often carried out during the light phase. While this might be more convenient for the experimentalist, it overlooks the fact that mice are nocturnal animals and that they mostly rest during the light phase and are active during the dark phase. Daily rest-activity rhythms are accompanied by pervasive daily oscillations in physiologic and metabolic parameters, including body temperature, blood pressure, heart and respiration rate as well as oxygen consumption (Adamovich et al., 2019; Douma and Gumz, 2018; Harfmann et al., 2015; Martino and Young, 2015; Reinke and Asher, 2019). Many of these parameters have been implicated in exercise capacity. Indeed, evidence from human and animal studies suggest that time of day is a critical parameter that should be taken into consideration when assessing exercise performance (Duglan and Lamia, 2019; Dyar and Eckel-Mahan, 2017; Gabriel and Zierath, 2019; Mirizio et al., 2020; Parr et al., 2020; Sato et al., 2019; Wolff and Esser, 2019).

Recently, we employed a treadmill running protocol during different times of the day and demonstrated that mice exhibit daily variance in their exercise capacity, primarily for medium and low intensity efforts (Ezagouri et al., 2019). Furthermore, by analyzing gene expression and metabolite composition in skeletal muscle, we uncovered potential modifiers of exercise capacity.

Thus, rodent treadmill exercise protocols performed at different time of day can serve as a powerful platform for identification and characterization of physiological and metabolic factors that alter exercise capacity and their underlying molecular mechanisms.

### Choosing your zeitgeber time

**Figure 1:**
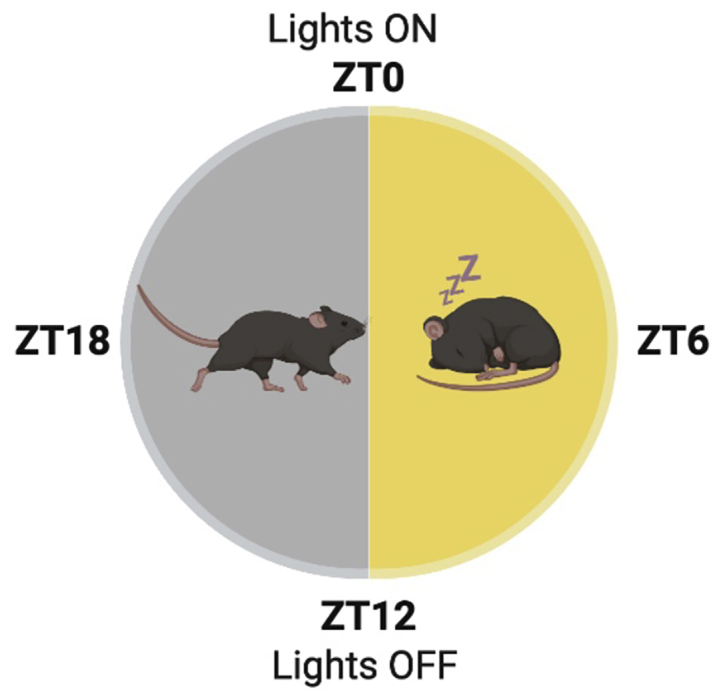
An illustration of ZT coordinates in conjunction with rest-active phases and light-dark schedule

Mice, as nocturnal animals, are mostly active during the dark phase and sleep during the light phase. In the absence of environmental timing cues (e.g., light-dark schedule), these sleep-awake cycles, as well as various other physiologic and metabolic rhythms, are maintained by an endogenous circadian clock (Ripperger et al., 2011). The circadian clockwork generates rhythmicity with a period of approximately 24 h. C57BL/6J mice, for instance, have an internal period of ∼23.7 h (Jud et al., 2005). These rhythms have evolved in adaptation to the daily rotation of the earth around its axis and are considered to provide fitness advantage. Circadian clocks respond to external signals, and are thus repeatedly synchronized by environmental cues and align themselves with the geophysical time. Light is the most potent external timing cue, or “zeitgeber” (“time giver” in German) for the circadian system, specifically for the master clock which resides in the Suprachiasmatic Nucleus (SCN) in the brain (Dibner et al., 2010; Golombek and Rosenstein, 2010). Laboratory mice are housed in animal facilities usually under a light schedule of 12 h light and 12 h dark (LD 12:12). This serves as coordinates to express zeitgeber time (ZT), where, by convention, ZT0 corresponds to lights on and ZT12 to lights off (Figure 1). As mentioned above, the time of day should be carefully considered when performing exercise tests. Regardless of whether the protocol is performed in a specific time of day or at multiple times, one should report the specific ZT at which the experiment was conducted. It is also recommended to continue with the same light-dark schedule during the protocol to avoid circadian disruption or excessive stress to the animals. For example, a protocol that started at ZT4 will be performed under light conditions, while one that started at ZT16 will be performed in darkness with the aid of dim red light. Although we now realize that exposure to red light may have some effects on circadian rhythms, it is still considered the best option for minimizing circadian disruption (Dauchy et al., 2015; Zhang et al., 2017).

### Acclimatize mice under a light-dark cycle that suits your experimental design

**Timing: 2 weeks**

**Figure 2:**
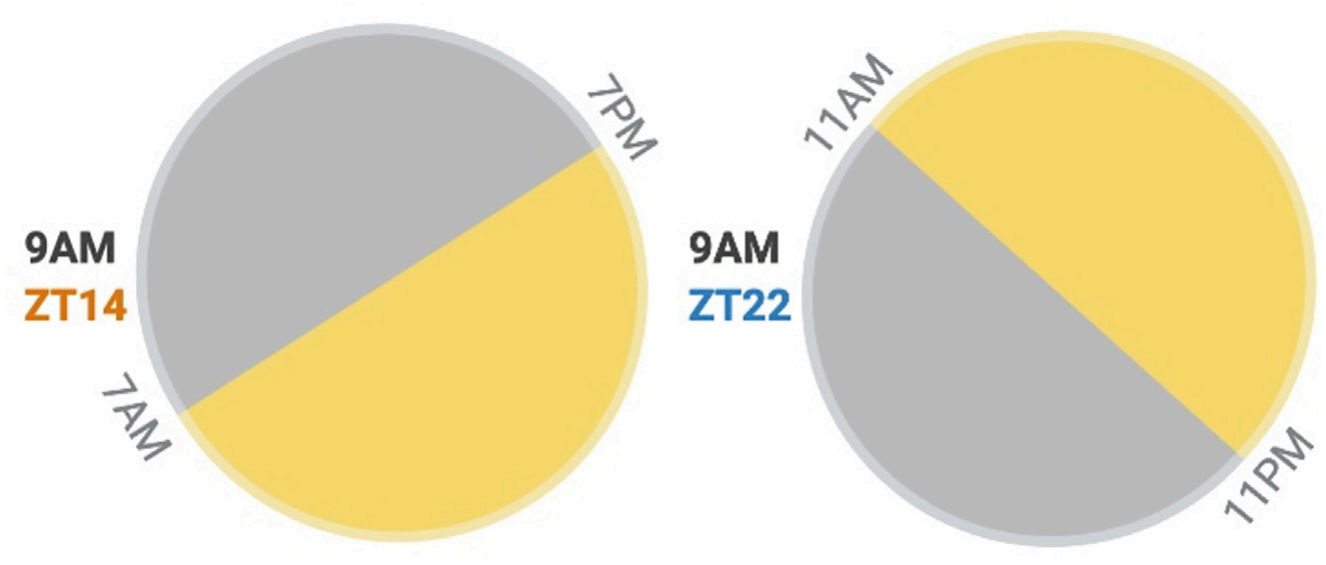
An illustration of using different light-dark schedule to allow performing the protocol at two different ZTs simultaneously

If you wish to perform the protocol during the active phase of the circadian cycle but rather not work in the middle of the night, light-dark cycles can be shifted to match your preferable working hours. One option, used by some animal facilities, is to have an additional room with an inverted light-dark cycle. For example, if ZT0 and ZT12 are normally at 8AM and 8PM respectively, after inverting the light-dark cycles, ZT0 will be now at 8PM and ZT12 at 8AM. The use of light controlled circadian cabinets provides even greater flexibility as you can schedule the light regimen in each chamber according to your needs. The latter allows you to perform the protocol in different ZTs simultaneously in a single convenient time (Figure 2).

**CRITICAL:** These adjustments must be carefully designed and arranged in advance to provide sufficient time for the circadian clock to align and animals to adjust to the new light-dark schedule. Adaptation to the new light regimen can last between 2 and 12 days depending on the magnitude (the time-difference between the old and the new light schedule) and directionality (i.e., delayed or advanced in respect to the old schedule) (Kori et al., 2017).

1. Choose the number of animals and conditions according to your experimental question.
2. Select a preferable time of day to perform the protocol (e.g., 9AM). Take into account your working hours and the total duration of the protocol.
3. Decide on the ZT at which you will start the protocol (e.g., ZT14).
4. Synchronize the mice to the appropriate light-dark schedule (e.g., to perform the protocol at 9AM and at ZT14, the light-dark schedule should be: lights on at 7PM, lights off at 7AM).
5. Preferably, wait at least two weeks before starting the protocol to ensure that mice are fully aligned to the new light-dark schedule.

### Handle mice to reduce variability

**Timing: ∼3 days**

To minimize stress associated with interaction with humans, mice need to be familiarized with human touch (Bailey, 2018; Deacon, 2006; Ghosal et al., 2015). We found that gentle handling of the animals prior to the experiment reduces fluctuation in blood glucose levels during the run as well as variability in performance within the same experimental group.

6. Gently pick up the mouse by the tail.
7. Place the mouse on the wire lid or on your coat and allow the animal to get a grip.
8. Massage the mouse tail from the base to the tip several times for 30 s.
9. When you are done, return the mouse to the cage.

***Note:*** Perform this practice routinely for several days prior the run (we normally do it for three consecutive days), preferably at the same time the mice are scheduled to run.

### Setup your treadmill

**Timing: 15 min**

10. Ensure the treadmill is on a flat surface using a level meter.
11. If you wish to include a slope, set it up to the desirable angle, usually 5°–10° (Conner et al., 2014; Dougherty et al., 2016; Marcaletti et al., 2011), using the treadmill device panel or with the aid of a digital goniometer.

***Note:*** We regularly perform the protocol without a slope, making it easier for mice to run. Adding inclination is energetically more costly and requires more power and, therefore, should be considered based on your experimental design and goals.

12. Adjust the electric shock intensity to 0.4 mA.

***Note:*** Many studies use rodent treadmills equipped with shock grids that stimulate mice to run (Dougherty et al., 2016; Fan et al., 2017; Narkar et al., 2008). These shocks are mild and are probably more disturbing than painful. Yet, some researchers prefer using a less stressful procedure to encourage their mice to run (see limitation section).

13. Make sure the speed is set to 0 cm/s.
14. Use the red light if you chose to perform the experiment in the active (dark) phase of the animals.

**CRITICAL:** Before you start with the experiments your protocol should be approved by your institutional Animal Care and Use Committee (IACUC).

## Key resources table

**Table:** 

REAGENT or RESOURCE	SOURCE	IDENTIFIER
**Other**		

Glucometer	Contour BAYER	N/A
Glucometer test strips	Contour Test Strips	50320933
5 lane treadmill	Panlab Harvard Apparatus, Barcelona, Spain	N/A
Plastic Leveler Torpedo ruler water level meter	HACCURY	YK-C1503314
Curved-end forceps	Bar Naor	BN 13-035-10BB
BD Precisionglide syringe needles, gauge 25, L 1 inch	Sigma-Aldrich	Z192406
Surgical scissors	Bar Naor	BN11-440-09ASC

**Experimental models: organisms/strains**		

12- to 14-week-old C57BL/6J male mice	HARLAN Biotech Israel Ltd.	N/A

**Deposited data**		

Transcriptome profiling	Gene Expression Omnibus database (GEO)	ID: GSE117161
Metabolomics data	MetaboLights database	Accession number: MTBLS145

## Step-by-step method details

Here we detail a protocol that applies a constant running speed at a moderate intensity, which is often referred to as “endurance running.” Endurance running, or long-distance running, is a type of exercise that involves aerobic metabolism. The running intensity is defined by the speed and slope that corresponds to the “aerobic power” needed. A moderate intensity run is often considered as 45%–75% of the maximal running capacity (Hawley et al., 2014). To determine the maximum power, you need to perform a treadmill test together with gas analyzer equipment and obtain the VO_2_max/VO_2_peak (Hoydal et al., 2007; Petrosino et al., 2016). A simpler, yet less accurate, alternative is to do an incremental speed test until mice cannot run anymore and obtain the maximum velocity (Vmax), which correlates with VO_2_max/VO_2_peak (Jordan et al., 2017; Picoli et al., 2018) . In this protocol we use a speed of 22–25 cm/s which is considered as moderate intensity (within ∼60% of their maximal velocity), as untrained C57BL/6J mice reach a Vmax on average at 40 cm/s (Ezagouri et al., 2019). The moderate intensity running session lasts a few hours, depending on the time of the day, until animals reach fatigue (see below).

***Note:*** the duration of this protocol is different than an acceleration speed protocol that lasts a few minutes, until mice are unable to maintain the elevated running speed and increased workload, which is accompanied by elevated blood lactate levels, and often referred to as “run to exhaustion.”

**CRITICAL:** As oxygen and energy needs differ relative to body size and composition, make sure you record the bodyweights in your study and preferably use weight-matched animals.

### Habituate mice to run on the treadmill

**Timing: 30 min × number of days needed**

Habituation period is needed for mice to get familiar with the treadmill and the running protocol. During this period, mice learn to avoid the shock grid. It was previously reported that for C57BL/6 mice, one to two short (5–10 min) sessions at low speed (15–17 cm/s) are enough to minimize their contact with the grid (Dougherty et al., 2016; Marcaletti et al., 2011). We, therefore, use a single habituation session the day before the test when working with C57BL/6J mice. However, additional familiarization sessions might be required for other mouse strains and depend as well on their age, and health status (see: Troubleshooting: Problem 1). When the purpose is to evaluate running capacity in sedentary non-exercised mice, it is also recommended to minimize as much as possible any training effects on performance. This can be achieved by using relatively lower speed. In case more habituation sessions are needed, scheduling these sessions every other day over a week may be considered (Castro and Kuang, 2017; Fan et al., 2017).

1. A day before the test, mice are acclimatized to the treadmill at the same ZT of the running test.

***Note:*** Exercise bouts were shown to affect the muscle molecular clock depending on the time of day (Wolff and Esser, 2019; Wolff and Esser, 2012). Therefore, performing the habituation sessions and the running tests at the same ZT are likely to reduce related confounding effects.

**CRITICAL:** If you perform the experiment within the dark phase work under red light. If necessary, use black cloth to cover the cages while transferring the animals to the treadmill.

2. Gently pick up the mice by the tail and individually place them in separate lanes.
3. Allow them to freely explore it for 5 min (speed 0 cm/s; shocks on)
4. Turn on the treadmill belt and increase the speed to 12 cm/s.
5. Increase the speed by 1 cm/s every minute until reaching 22 cm/s.
6. Continue running mice at 22 cm/s for 5 min.
7. When you are done, return the mice to their cages.

***Note:*** you might find it easier to pick up mice by the tail from the treadmill while the belt is still running.

### Run mice at a moderate intensity to evaluate running capacity

**Timing: variable; few hours**

To reliably determine running capacity, the endpoint as well as inclusion and exclusion criteria should be well defined. The challenge is to decide on when the mice reach fatigue, and as discussed below (see limitation section) one can find different protocols in the literature. Based on early studies from the Evan’s lab (Fan et al., 2017; Narkar et al., 2008) and our experience, we defined two criteria for fatigue, and observing either of them is an indication to stop the run and return the animal to its cage. At the beginning of the test, sedentary C57BL/6J usually cope well with the speed and run toward the front of the belt. After few hours, they spend more time at the rear of the treadmill belt and blood glucose levels progressively decline (Methods Video S1). Hence, the first criterion is reaching blood glucose levels of ≤70 mg/dL. We did notice, however, that in the case of some mutant mouse models (e.g., *Per1/2* null mice (Ezagouri et al., 2019)), after running a certain distance, the animals are reluctant to run and spend a considerable time in contact with the gird, while their blood glucose levels remain above 70 mg/dL. We, therefore, included a second criterion, namely spending more than 5 accumulative seconds in contact with the grid within a 30 min window after at least 1 h run.

***Note:*** In the case of C57BL/6J, if mice spend more than 5 accumulative seconds in contact with the grid within a 30 min window already within the first hour of the run, they are excluded from the experiment. (Troubleshooting: Problem 1)

**Figure 3:**
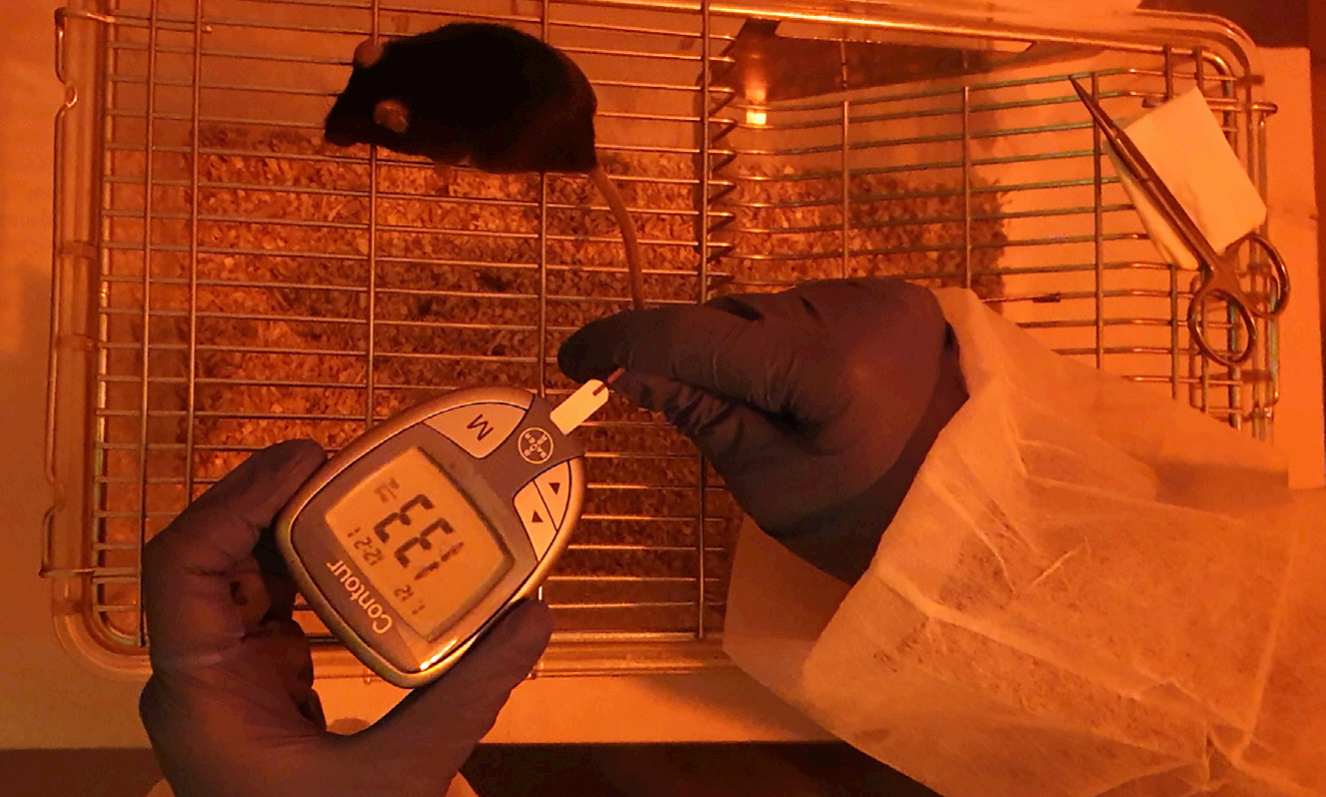
An example of glucose monitoring from a mouse tail tip
  g. Apply gentle pressure with dry paper towel or gauze for approximately 10 s to stop the bleeding.

**Figure 4:**
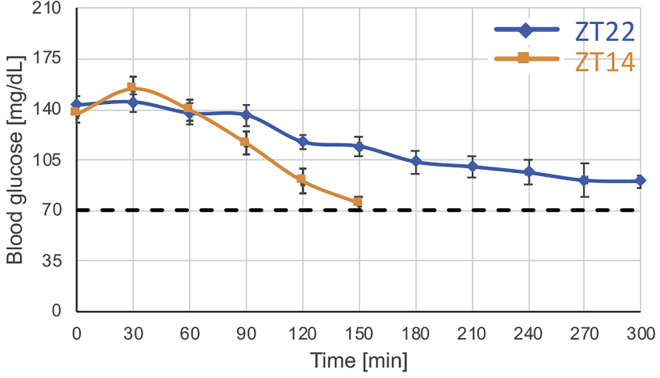
Expected blood glucose levels during moderate intensity (25 cm/s) running test performed at two different ZTs during the active phase Data are presented as the mean ± SEM of n = 3. p < 0.05, p < 0.01, p < 0.001 repeated measures two-way ANOVA for the interaction, ZT effect, and running duration effect, respectively.

8. If you perform the experiment in the active (dark) phase work under red light. If necessary, use black cloth to cover the cages while transferring them to the treadmill location.

9. Measure the initial blood glucose levels by using glucometer:
  a. Get supplies ready for use:
    i. Prepare sharp and clean scissors
    ii. Moisten a small piece of paper towel or gauze with water or 70% ethanol
    iii. Place glucose strip into the glucometer
  b. Gently remove the mouse from the cage and record its weight.
  c. Pick up the mouse by the tail and place it on the wire lid or on your coat to allow it to get a grip.
  d. Keep holding the mouse with your non-dominant hand and cut 1–2 mm from the tail tip.
    ***Note:*** If prior tail biopsies have been performed (e.g., for genotyping purpose) lateral tail vein incision will suffice.
  e. Gently massage the tail from the center of the tail to the tip to allow for blood flow.
  f. Place the blood sample on the glucometer strip and record the results (Figure 3).

10. Place the mouse in the lane.
11. Repeat for all mice that are expected to start the run.
12. When you are done start the free exploration session (5 min; speed 0 cm/s; shocks on)
13. Turn on the treadmill belt and increase the speed to 12 cm/s, then increase the speed by 1 cm/s every minute until reaching 25 cm/s.
14. Continue running mice at 25 cm/s until they reach fatigue according to the criteria describe above.

15. Take mice for blood glucose measurements every 30 min to monitor fatigue status. Usually, a rise in blood glucose levels appears during the first 30 min, but overall glucose levels are expected to gradually decline throughout the test (Figure 4).
  a. Take one mouse at a time gently and quickly pick it up by the tail.
    **CRITICAL:** Keep the treadmill belt running while picking up a mouse. It is faster and easier to grab the mouse while it is still running. This way you do not disturb the running of the other mice while you take the necessary measurement.
  b. Place the mouse on a wire lid or on your coat to allow it to get a grip.
    Use wet paper towel or gauze and gently remove the scab.
    **CRITICAL:** Removing the scab and allowing fresh blood flow by massaging (“milking”) the tail is important to get reliable glucose reads.
  c. Place the blood sample on the glucometer strip and record the results (Figure 3).
  d. Once you are done place the mouse back in its lane.
    ***Optional:*** The protocol can be modified depending on your experimental question:
    i. To induce training effect, use the protocol for shorter durations (e.g., 30–60 min) several days a week for a few weeks (Ferreira et al., 2007; Jordan et al., 2017; Massett and Berk, 2005; Narkar et al., 2008).
    ii. For tissues harvesting for further processing and analysis, stop the protocol at a pre-defined time and sacrifice the mice. (Troubleshooting: Problems 1^2^,3,4)

Methods Video S1. A video of mice running on treadmill, related to step 14 Compare left and right lane: on the right lane a mouse that can sustain the intensity of the run, and on the left lane a mouse that shows fatigue signs.

### Dissecting soleus, gastrocnemius plantaris, and quadriceps muscles for further analyses

**Timing: 10–30 min**

Here we describe how to dissect different types of skeletal muscle for further analysis, comparing running versus sedentary conditions. This is valuable if you wish to study molecular changes occurring in skeletal muscles during the running session, once animals reach fatigue, or even later on upon recovery.

***Note:*** It is recommended to recognize the structure of the muscles and get familiar with the relevant anatomy. Briefly, muscles are connected to bones through tendons, and to the body by connective tissue, therefore make sure you dissect a muscle that is clean of irrelevant tissues as much as possible.

**CRITICAL:** There are two valid options for selecting the sedentary control group. Given that the run can last for several hours, and that this time duration by itself might lead to some metabolic/physiologic changes this should be taken into consideration. Hence, you can either sacrifice the control group at the time that corresponds to the start or the end of the run. For example, in the case of a mouse that started the running protocol at ZT14 and run for 3 h. One option is to have a similar end point for the sedentary control group, i.e., ZT17. In this case, the sedentary control group are placed near the treadmill in individual cages without food and water, which is as close as possible to the running conditions. You can also sacrifice the control group at a separate day after keeping the mice in the treadmill’s lanes without turning on the running belt for the same duration as the running group. Another option is to sacrifice the control group at ZT14 to get a picture of the condition before the run. It is recommended, therefore, to take this into consideration when designing the experiment and interpreting the results.

16. Sacrifice the mouse at the desirable time after running.

***Note:*** We found that cervical dislocation without anesthesia is the preferable technique as it is relatively rapid and enables sacrificing several animals within a short time. Nevertheless, cervical dislocation can damage blood vessels, resulting in internal hemorrhaging. Therefore, this technique is not recommended if a large volume of blood is needed for future analysis, and alternative techniques should be considered.

**Figure 5:**
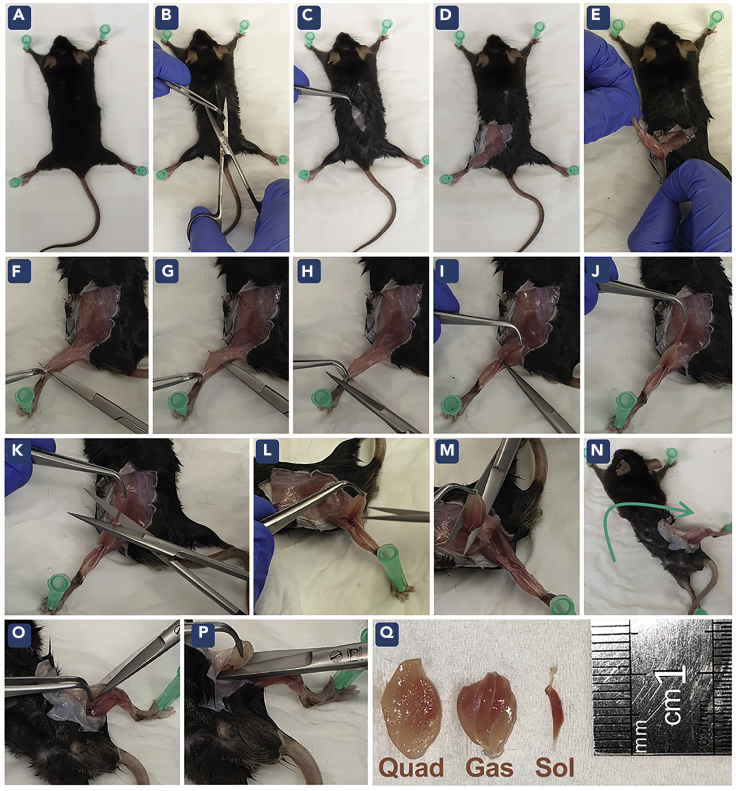
Dissection of mouse soleus (Sol), gastrocnemius plantaris (Gas), and quadriceps (Quad) muscles

***Note:*** At this stage you can freely work under light.

17. Immediately after the mouse is sacrificed, lay it on the dissection board in a prone position. Stabilize its feet with needles / tapes, and spray ethanol on the lower body until the fur becomes wet (Figures 5A and 5B).

18. Use the forceps to lift the skin around the distal part of the spine (In the middle of the transverse line between the two points where the hind limbs are connected with the body) and make an incision using a pair of scissors (Figure 5C).
19. Use the forceps to lift the skin around the incision side that is closest to the hind limb. Insert the scissors between the uplifted skin and the body and make an opening move with the blunt side of the scissors touching the internal side of the skin to enlarge the incision toward the posterior side of the hind limb (over the hamstring and the gastrocnemius muscles). Cut the skin gently with the scissors each time the incision is large enough until the Achilles tendon is exposed (Figure 5D).
20. After the Achilles tendon is exposed, gently hold the skin of the anterior part of the distal hind limb with your fingers then peel the skin of the mouse hind limb until it is completely visible (Figure 5E).
21. Use the forceps to pick up the distal part of the Achilles tendon, and hold it firmly. Using the second hand, stick the point of the scissors between the spot where the Achilles tendon is connected to the gastrocnemius muscle (the proximal end part of the Achilles tendon) and the tibia / fibula bones. The scissors should stick to the bones to create a space between the muscle and the bones. Keeping the scissors closed, slide them along the bones and toward the knee until you reach the patella. If performed correctly the gastrocnemius plantaris and soleus are now separated from the bones (Figure 5G, Methods Video S2).

22. Using the forceps, hold the proximal edge of the Achilles tendon, then cut distal to it. Make sure you cut the tendon in its distal end in order to avoid the cutting of the distal part of the soleus (Figure 5H, Methods Video S2).
23. Keep holding the hind limb muscles by the tendon (Figure 5I, Methods Video S2).
24. Locate the soleus muscle, and use scissors to cut the proximal end of the soleus tendon. If performed correctly, the soleus will pop up (Figure 5J, Methods Video S2).
25. Use the forceps to hold the proximal part of the soleus (the detached part), gently pull it up so the whole muscle can be clearly observed. Cut the distal tendon to completely isolate it. Put the dissected muscle inside a tube and immediately freeze in liquid nitrogen (Figure 5K, Methods Video S2).
26. Use the forceps to hold the Achilles leftovers (on the distal part of the gastrocnemius plantaris) in parallel to the bones, using the scissors in the second hand cut the non-muscle tissue around the proximal part of the gastrocnemius plantaris, along the bones until the muscle is fully detached. Use the scissors to clear the muscle of any non-muscle tissues. Put the gastrocnemius plantaris inside a tube and immediately freeze in liquid nitrogen (Figures 5L and 5M).

***Note:*** The plantaris muscle is synergist to soleus and gastrocnemius, but more similar to gastrocnemius in terms of fiber type composition and metabolic profile (Sawano et al., 2016). The plantaris is enmeshed within the medial and lateral heads of the gastrocnemius muscle, and separating the two can be challenging. Due to its similarities to gastrocnemius and its significantly smaller mass we collect them together.

27. Cross the leg to the other side of the body (Figure 5N), and stabilize it to the dissection board using a needle so a 90 degrees angle of the knee joint is formed.
28. Use the forceps to pick up the quadriceps so a space is created between the muscle and the femur. Use the scissors to make a cut between the muscle and the femur, as close as possible to the bone. Then cut along the bone toward its proximal end until the muscle detaches (Figure 5O). Using the forceps, hold the distal part of the detached quadriceps, and with the scissors in the other hand, make a cut toward the proximal end of the muscle and along the bone until the muscle is linked to the body only by connective tissue (Figure 5P). Using the scissors, clear the muscle of any non-muscle tissue. Put the muscle inside a tube and freeze instantly in liquid nitrogen. (Troubleshoot: Problem 5)

Methods Video S2. A video showing dissection of the soleus muscle, related to steps 21–25

## Expected outcomes

We previously reported that 3 months old C57BL/6J mice exhibit daytime difference in their endurance running capacity (Ezagouri et al., 2019). Specifically, when mice were challenged to cover a 4.5 km distance (5 h at speed of 25 cm/s) using this protocol at ZT22 (end of the active phase), they were able to maintain blood glucose levels above 70 mg/dL. By contrast, the group of mice that run at ZT14 (beginning of the active phase), failed to run the entire distance, and their blood glucose levels dropped below 70 mg/dL much earlier (Figure 4, also see example in Methods Video S1).

A proper dissection will yield intact soleus, gastrocnemius plantaris, or quadriceps muscles as depicted in Figure 5Q. These tissues can be frozen and stored at −80°C until further analyses, such as gene and protein expression as well as metabolites content.

## Limitations

### Use of shocks as aversive stimulus

As opposed to voluntary wheel running activity, forced treadmill running is stressful, requires negative stimulus and direct intervention from the experimenter. Yet, it provides control of time, intensity, and duration. Although mice are naturally prone to run, an external motivator is required to keep them performing the running treadmill test. To encourage running we suggest here to use a light electrical shock stimulus, an option available in various commercial treadmills. A metal grid is located at the end of the running belt and provides mice with electrical shock, in response to which they continue to run. In most cases, mice learn to avoid this zone during the training session performed a day before the running test. For ethical and humane reasons, some researchers prefer to use other motivation strategies that rely on tail tickling (Bouganim and Bergdahl, 2017; Conner et al., 2014; Duglan et al., 2020; Li et al., 2007). These include directing short puff air using a compressor, installing soft brushes, or a plastic curtain at the back of the treadmill. It is noteworthy, that manually pushing the mouse onto the treadmill by touching or pocking the mice with the hand or any other tool might introduce variability and bias, as the force and frequency used depends on the experimenter, and therefore should be applied cautiously. Conceivably, different motivation strategies affect the running performance. This needs to be carefully considered when designing the experiments and comparing results with existing data in the literature.

### Differences between protocols in the literature

There are numerous treadmill running protocols for mice in the literature, making it very challenging to prescribe one. In fact, there is no consensus on whether to add a slope or not, what slope angle to choose, and whether to start with continuous ramp or incremental speed. Exhaustion criteria and motivation techniques, as stated above, are also heterogenous. This is probably because protocols and terminology are often adopted from human running performance tests and their implementation to mice sometimes vary. Therefore, it is critically important to provide a detailed protocol of the test performed alongside with the time of the day when reporting your experiments and also take it into consideration when comparing your data with existing reports in the literature. Notably, in some reports, authors do not always specify the time at which the experiment was performed, making it difficult to compare the results. In other cases, while the specific hour of when the protocol started is reported, the light-dark cycle is simply written as “12 h light-12 h dark.” This is meaningless unless you specify the times lights were turned on and off. Therefore, as explained above, we recommend using the term ZT to indicate the time.

### Strain, age, and sex differences

Running capacity and exercise performance in general, are affected by physiological variables that are not only shaped by the time of day, but also by genetic background, sex, and age (Avila et al., 2017; Billat et al., 2005; Kemi et al., 2002; Lerman et al., 2002). It is, therefore, good practice to consider all the above parameters while designing your experiment and interpretating the results.

## Troubleshooting

### Problem 1

Poor performance at an early stage of the run (steps 1–7)

Some types of mice might frequently spend more time in contact with the shock grid or otherwise perform poorly (e.g., running in the opposite direction several times).

### Potential solution

In animals in which the problem is frequent, additional habituation sessions are recommended and depend on the mouse strain, genetic model, age, and health condition (e.g., obese, cardiovascular, and neurodegenerative diseases).

### Problem 2

Glucose measurements are inconsistent with previous results or throughout the test (steps 14 and 15)

### Potential solution

Test, the treadmill parameters, (speed, slope, and shock), check the glucometer by comparing it with another apparatus. Inquire whether there were any problems with the light-dark schedule prior to the run that might have shifted the circadian clock.

### Problem 3

High variability in performance of mice from the same cohort (steps 14 and 15)

### Potential solution

Check mice housing conditions, light-dark schedule, genotype, and treadmill parameters (speed, slope, and shock).

### Problem 4

Mice do not reach the endpoint criteria within a reasonable time frame (steps 14 and 15)

We observed that trained mice can perform the test for more than 5 h, this may also occur with different mouse models, or under various treatments.

### Potential solution

The duration of the protocol can be shortened by increasing the treadmill speed or adding an inclination.

### Problem 5

Dissecting the wrong muscle (steps 21–28)

Differentiating between specific hind limb muscles can be challenging for a non-experienced experimenter. Extra caution should be used when separating the soleus and the gastrocnemius plantaris from the tibia and fibula bones, and when cutting the distal end of the Achilles tendon in order to avoid cutting the soleus tendon (steps 21 and 22). When inappropriately separated, the soleus will stick to the gastrocnemius plantaris, making it difficult and sometimes impossible to separate them.

### Potential solution

Make sure you get familiar with the relevant anatomy, and are able to recognize the structure of the above-mentioned muscles in particular. Practice the procedure multiple times in a pilot experiment before performing the experiment of choice.

## Resource availability

### Lead contact

Further information and requests for resources and reagents should be directed to and will be fulfilled by the lead contact, Gad Asher (gad.asher@weizmann.ac.il).

### Materials availability

This protocol did not generate any unique reagents.

### Data and code availability

This protocol did not generate any unique codes.

The experiments conducted by Ezagouri et al. (Ezagouri et al., 2019) yielded the following datasets: (1) Transcriptome profiling was deposited in the Gene Expression Omnibus database (GEO) under the ID code: GSE117161. (2) Metabolomics data have been deposited in the MetaboLights database under accession number MTBLS145.
